# Sensor-based evaluation of a Urine Trap toilet in a shared bathroom

**DOI:** 10.1016/j.scitotenv.2022.159178

**Published:** 2023-01-20

**Authors:** Prateek Kachoria, Sarani Sasidaran, Claire M. Welling, Praveen Rosario, Jin Zhou, Krishnendu Chakrabarty, Harald Gründl, Lotte Kristoferitsch, Sonia Grego

**Affiliations:** a RTI International India, New Delhi 110 037, India; b Center for Water, Sanitation, Hygiene and Infectious Disease (WASH-AID), Department of Electrical and Computer Engineering, Duke University, Durham, NC, USA; c Department of Electrical and Computer Engineering, Duke University, Durham, NC, USA; d EOOS Design GmbH, Zelinkagasse 2/6, 1010 Vienna, Austria

**Keywords:** User testing, Nitrogen pollution, Source-separation toilet, Urine diversion, Time series analysis

## Abstract

Urine diversion in a No-Mix Toilet is a promising approach for sustainable fertilizers and reduction of the nutrient load for wastewater treatment; however, user adoption remains a challenge. This study evaluates the Urine Trap, a passive No-Mix toilet design based on the teapot effect, wherein the urine stream inlet is invisible to the user and therefore it does not impact the user experience for increased adoption.

This study evaluated the nutrient separation performance of a Urine Trap flush toilet in a bathroom shared by women in two sites in India. Over three different testing periods, 841 uses of this squat plate were recorded in 50 days. Analytical measurements found 36 % separation efficiency for total nitrogen (TN). While effective, the Urine Trap under test by users did not yield a 70–80 % TN separation efficiency observed under engineering characterization. High temporal resolution data from sensors on waste collection tanks, the opening of the bathroom door, and cleansing water flow were used to gain insights into hygiene practices. The data showed a frequent habit of wetting the squat plate during physiological excretion, a hygienic practice that eases cleaning but degrades the teapot separation effect of the Urine Trap design. By using sensors, we demonstrate a method to non-invasively gain quantitative insights into hygiene practices to inform sanitation technologies deployment strategies for improved outcomes.

## Highlights

• No-Mix toilets remove nitrogen from wastewater to facilitate treatment and sustainability.
• Urine Trap is a No-Mix design wherein the urine stream inlet is invisible to the user.
• User testing found the Urine Trap passively removes 36 % of the nitrogen (TN).
• Sensor-based wastewater patterns reveal hygiene practices impact TN separation.

## Graphical abstract

**Figure:**
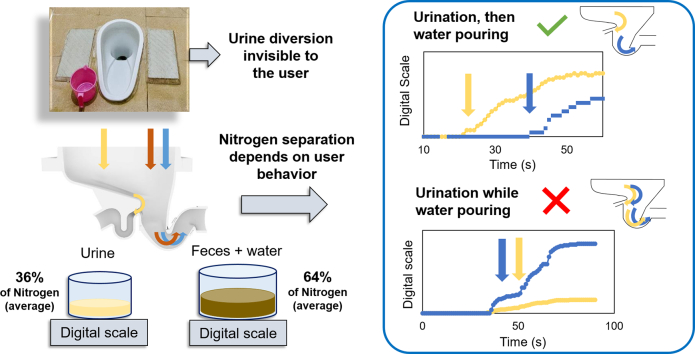

## 1 Introduction

Human excreta contain the same nutrients (such as nitrogen and phosphorus) as the fertilizers used for food production (Senecal and Vinnerås, 2017). While fertilizers are produced with energy-intensive and polluting processes, human excreta end up as untreated waste for billions of people, and the lack of safely managed sanitation creates significant health and environmental problems (WHO/UNICEF, 2019) (Nazari et al., 2020). In response to the global challenges of improved sanitation and sustainable agriculture, a body of research has gone into pursuing nutrient recovery from waste streams. Among the waste streams, human urine provides an attractive potential platform for agricultural resource recovery, especially nitrogen (Wald, 2022). Furthermore, since urine represents only 1 % of wastewater volume but 80 % of the wastewater nitrogen, removing urine from wastewater enables treatment at reduced cost and energy (Tarpeh et al., 2018).

A major challenge to enhance efficiency of resource recovery using urine is to avoid water dilution associated with conventional wastewater generation, since Total Nitrogen (TN) content in urine (0.7 %) is already much more diluted than in fertilizer (46 %) (Udert et al., 2006). Source separation at the toilet level to prevent urine from mixing with other wastewater in urine-diversion or No-mix toilets has been proposed as an attractive solution (Larsen et al., 2009).

Typically No-Mix or urine diversion technologies comprise of a custom toilet that has two compartments for urine and feces, respectively, and a dedicated conduit to transport separated urine to treatment (Nazari et al., 2020). No-Mix toilet designs include waterless or dry toilets, which are often deployed in the global south but are undesirable due to fouling or malodor (Gounden et al., 2006; Mkhize et al., 2017), unless novel sophisticated designs are employed (Hennigs et al., 2019; Hennigs et al., 2021).

Urine diversion flush toilets were favorably viewed in a European study for their nutrient recycling purpose (Lienert and Larsen, 2010); however, major barriers to consumer adoption were found in systems that were cumbersome and unreliable.

EOOS Design (Vienna, Austria) has developed a novel No-Mix flush toilet design, the Urine Trap, that overcomes these barriers and achieves passive separation of urine and water flush with a system with the same appearance for the user (Gundlach et al., 2021). This Urine Trap design takes advantage of the so-called teapot effect described as the attachment of liquids to a curved wall due to surface tension. This effect is used to efficiently guide the urine along a curved entrance invisible to the user and into the collector. Laboratory results indicate that the optimized design has only 2.5 % of flush water in the urine collection and a urine separation efficiency ranging from 60 % to over 95 % (Gundlach et al., 2021).

The Urine Trap design was adapted to a squat plate, a toilet design commonly adopted in South Asia, including India. South Asia is a region with a large sanitation gap and where decentralized sanitation is common and where there is an urgent need for onsite technologies to treat wastewater to a high standard of pathogen, organic and nutrient removal (Krithika et al., 2017; Winkler et al., 2017; Welling et al., 2020; Dasgupta et al., 2021).

The operation of a Urine Trap toilet designed for South Asia, a washing culture where water instead of toilet paper is used for personal cleansing, leads to potential challenges: while toilet paper clogging is no longer an issue, the fluid dynamics of water for personal wash is likely to degrade the separation performance and dilute the separated urine.

This study is a field testing evaluation of the Urine Trap toilet in a washing setting. The manuscript reports methods and results of the evaluation of a prototype Urine Trap flush squat plate in two bathroom facilities shared by women in India. The study was conducted with women users to eliminate the variable of gender-specific urination practices, namely urinating while standing (Schelbert et al., 2021).

We conducted a detailed analytical characterization of the two waste streams, urine and feces+ flush water, that were generated by the Urine Trap squat plate, and we determined the separation efficiency by volume and by nutrients (TN and total phosphorus). Analysis of wastewater generation temporal dynamics provided insights into user behavior, including washing practices that are bound to impact the nutrient separation performance. This paper describes the performance of a Urine Trap Indian toilet and a method to non-invasively determine user behavior in toileting to inform and optimize technology development and deployment.

## 2 Methods

### 2.1 Ethics

The study installed a test squat plate in one of multiple stalls in women-shared toilet facilities. The users of these facilities had free, unmonitored access to the test stall as well as other stalls. No user was identifiable by the collected data. The institutional review board (IRB) of PSG College Institute for Medical Science and Research reviewed the protocol (PSG IHEC 20/200) and exempted it from ethical review; the Duke University IRB also reviewed the protocol and deemed it not being human subject research. The community of users was aware that the sanitation facility was used to evaluate toilet technologies and were verbally informed that the specific toilet stall was under test by a female staff at the beginning of data collection. The users had uninterrupted access to alternative conventional toilet stalls because they could select the stall in this toilet facility as well as access additional nearby bathroom facilities.

### 2.2 Test sites and users

The Urine Trap toilet evaluated by this study was a drop-shaped squat plate designed for pour-flushing, i.e., it was not to be connected to a cistern. Two identical Urine Trap toilet prototypes were installed in two shared-female bathrooms in the city of Coimbatore, India.

Data was collected in three separate time periods: period 1, 14 days in August–October 2020 and period 2, 20 days in February – April 2021 were collection at Site A. Data for period 3, 16 days in August – September 2021, was collected at Site B.

Both Site A and Site B were custom-built facilities for testing toilet technologies. The sites feature unmonitored user access to bathroom stalls and a separate space and entrance for engineering monitoring and sampling from the measurement system connected to the toilet waste streams. The onsite research team included one female engineer that could access the toilet area for testing purposes.

Importantly, the two facilities featured elevated bathroom floors with heights of 1.2 and 1.5 m for site A and B, respectively, below the toilet area in order to feed wastewater effluent by gravity to equipment located directly underneath the bathroom floor.

Site A was a privately-owned textile mill with approximately 20 resident workers on a term contract and 50 day workers. The test toilet facilities (described in Welling et al., 2020) included 10 bathroom stalls, one of which was used to install the Urine Trap toilet, and a separate handwashing sink with four taps.

Site B was a shared-toilet facility located on a college campus in proximity of a parking lot and outdoor break area, and was typically used by college staff. The Site B bathroom facility featured four bathroom stalls, one of which was used to install the Urine Trap toilet, and there was a separate handwashing sink with two taps.

**Fig. 1:**
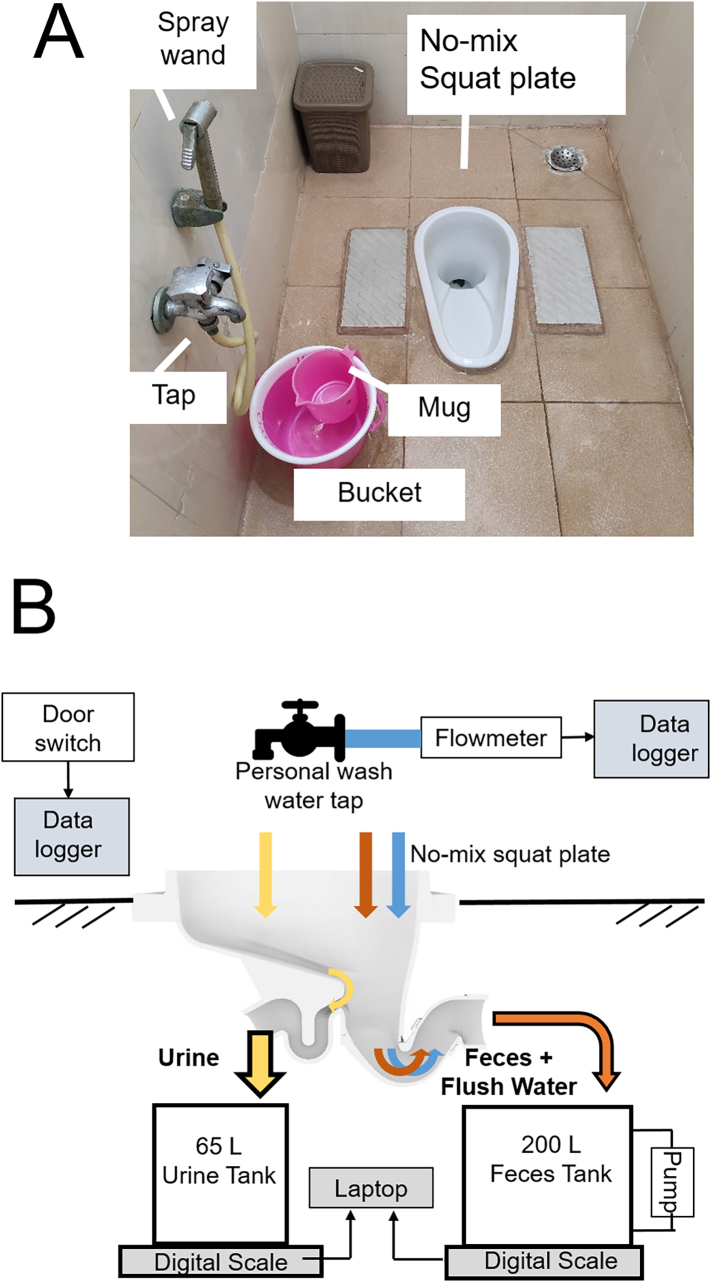
(A) Pictures of bathroom. (B) Schematic of the sensor-based data collection system.

For both sites, the toilet stall features a water faucet tap, both a mug and spray wand for washing, and a bucket for flushing (Fig. 1A).

The test stalls were open during the daytime with a caretaker in service and closed outside of business hours. Waste collected during work hours was allowed to settle overnight prior to sampling.

### 2.3 Sensors for measuring wastewater volume generation

The front and back exit holes of the squat plate were connected by PVC pipes to two HDPE tanks, namely the urine tank (UT) and feces tank (FT) placed on digital scales with an RS232 interface (Fig. 1B).

The urine tank (capacity 65 L) was placed on a 200 kg capacity scale (model EBPH12, 10 g resolution, 400 × 400 mm pan size, Everest Scales, India). The feces tank (capacity 200 L) was placed on a 300 kg capacity scale (model APC 300-K9, 20 g resolution, 600 × 600 mm pan size Everest Scales, India).

The plumbing between the squat plate and the inlet of the tank included a manual ball valve to direct the wastewater to either the sewer line or the tanks. The tank lids were not air-tight to avoid buildup of gases inside the tanks. Both tanks had an overflow connection to a sewer line.

Great care was taken to ensure that the plumbing did not perturb the ability of the scales to weigh incoming liquid nor cause any vibrations. The pipe going into the feces tank was flexible and independent of contact from anything other than the tank. The inlet pipe was rigid and supported from the wall and did not touch the tank (Figure supplementary S1.1 A, S1.2).

At the beginning of each day of wastewater data collection, both tanks were emptied to the sewer and rinsed and the scales were tared. The system received toilet water for the workday (typically 10 am to 5 pm) while continuously recording weight with 1 s resolution, and after that time the wastewater was diverted to the sewer. The weight was recorded using open source software, “Simple Datalogger” on a laptop and converted to volume using 1 kg = 1 L conversion, since the majority of the volume is water. Calibration of the digital tank scales was performed prior to data collection for each of the three periods of data collection and linearity from triplicate measures was high, with R^2^ = 0.99.

### 2.4 Water quality measurements

The Urine Trap toilet effluent streams were sampled from the urine tank and, using two different methods, from the feces tanks the day after collection.

The urine tank contents were homogenized by stirring with a long glass pipette and collected from a sampling port.

The feces tank collection took place after overnight settling so that the supernatant of the feces tank was collected through the sampling port. Then, a daily composite from the feces tank was obtained with a maceration pump (Jabsco 18950) installed in a recycling loop with the tank. The macerator operated at 45 LPM and ran for a time proportional to the volume in the tank and equivalent to 3 maceration cycles. After sampling, the macerator pump was also used to empty the feces tank into the sewer.

Sterilized 250 mL or 500 mL volume bottles were utilized for sample collection (Supplementary Fig. S1B).

The concentration in wastewater daily samples for the urine tank C_U_ and feces tank C_F_ was measured for the following parameters: Ammonia (NH3−N), Total Nitrogen (TN), Total Phosphorus (TP), Chemical Oxygen Demand (COD) by a DR-900 (Hach, USA) colorimeter using vendor kits. The kits were: for NH3-N Hach method 10,031 (range: 0.4–50 mg/L), for TN Hach method 10,072 (range: 2–150 mg/L), for TP Hach method 8190 (range 0.06–3.5 mg/L PO_4_^3−^), and for COD - Hach method 8000 (measuring range: 20–1500 mg/L).

Total suspended solids were measured as per TSS EPA method 160.2 and Total Solids following EPA Standard Methods section 2540 B. Electrical conductivity (EC) was measured with Myron L Ultra meter II.

The separation efficiency η of a parameter with concentration C, e.g. Total Nitrogen (TN), was a percentage measure of parameter concentration diverted to the urine tank, which was weighed by volume using Eq. (1)

(1) $$\;\eta \;\left(\%\right)=\frac{∆U*C_{U}}{∆U*C_{U}+∆F*C_{F}}$$

ΔU, ΔF: Volume collected in Urine, Feces tank (L) daily, C_U_, C_F_: Parameter concentration in Urine, Feces tank (mg/L). The separation efficiency for the supernatant of the feces tank was calculated using the same Eq. (1) and the C_F_ value measured from the supernatant.

### 2.5 Sensors for monitoring bathroom door and water use

As is customary in India, the bathroom included a water line that was used for personal cleansing. The water tap was used for filling a 5 L bucket that was used for both personal washing by a 1 L mug and for flushing the toilet. A spray wand for personal wash was also connected to the same tap to offer a hygienic alternative to the mug (Fig. 1A).

At the start of period 2, we began to record the water flowrate to the tap by an electro-magnetic flowmeter (FMG71B-A-BSP, Omega Engineering, USA) through a 4–20 mA datalogger (Hobo U120-006M, Onset Corporation, USA). The flowmeter was calibrated by collecting fixed volumes of 1 L, 2 L, and 4 L while recording the time to obtain flowrate. The measured flowrate and flowmeter output values were in agreement with R^2^ > 0.99. The water measurement apparatus and the scales of Site A were transferred to Site B for data collection in period 3.

In order to establish the duration of a bathroom visit, a magnetic door switch was installed at Site B. The door switch collected binary data indicating a change door status (open or close) and was connected to a pulse data logger (Hobo UX120-017, Onset Computer Corporation, USA).

### 2.6 Event determination and duration

A *use event* was defined as the use of the bathroom and generation of wastewater detected by at least one of the two digital scales. Within an event, a multiplicity of subevents could take place: two types of physiological subevents include urination and defecation, as well as user actions such as personal cleansing, by either the spray wand or the mug, and toilet flushing.

We treated the digital scale data collected for n seconds as a time series F ={*f*_1_, *f*_2_, …*f*_*n*_} from the feces tank scale and $U=\left\{u_{1},u_{2},\ldots u_{n}\right\}$ from the urine tank scale. The determination of each use event was carried out by adopting four steps: starting point detection, end point detection, event filtering, and event combination.

1. Start point detection. *ts*_*i*_ is the start time location of the *i*-th use event if (*f*_*t*+1_ − *f*_*t*_ > 0) ∨ (*u*_*t*+1_ − *u*_*t*_ > 0) for all *t* ∈ [*ts*_*i*_, *ts* + *th*_*s*_) where *th*_*s*_ is a parameter which depends on the scale response time to a change in weight and defines the time required for the scale reading to be changing to determine the start point.
2. End point detection. *te*_*i*_ is the end time location of the *i*-th use event if $\left(\frac{d^{2}F}{dt^{2}}\leq 0\right)\wedge \left(\frac{d^{2}U}{dt^{2}}\leq 0\right)$ for all *t* ∈ (*te*_*i*_, *te*_*i*_ + *th*_*e*_], where $\frac{d^{2}y}{dx^{2}}$ denotes the second derivative of *y* with respect to *x*, *th*_e_ defines the time required for the scale reading to be constant for the determination of an end point.
3. Event filtering. *i*-th event is recognized as noise and removed if changes in tank scales' weight are below pre-defined threshold, specified as (*f*_*te*_*i*__ − *f*_*ts*_*i*__ ≤ *th*_*f*_) ∧ (*u*_*te*_*i*__ − *u*_*ts*_*i*__ ≤ *th*_*u*_).
4. Event Combination. After all starting and ending points have been identified, two adjacent events (*ts*_*i*_, *te*_*i*_) and (*ts*_*i*+1_, *te*_*i*+1_) are combined as (*ts*_*i*_, *te*_*i*+1_) if the distance between them is below a pre-defined thresholds *th*_*c*_ where the distance is defined as *ts*_*i*+1_ − *te*_*i*_.

In this study, we selected the following empirically derived parameter values *th*_*s*_ = 10 *s*; *th*_*e*_ = 5 *s*; *th*_*f*_ = 0.05 gram; *th*_*u*_ = 0.05 gram; *th*_*c*_ = 60 *s*.

For each use event, we calculated its duration (*te*_*i*_ − *ts*_*i*_) as the feature for the classification of an event to be labelled as a urination or a defecation event. Since no ground truth data on the labels was known, the unsupervised method Gaussian Mixture Model (GMM) was used to cluster the detected events into two groups (Reynolds, 2009). Note that GMM is a probabilistic model based on a linear combination of *K* Gaussian distributions, as shown in Eq. (2):

(2) $$p\left(x\right)=\sum_{i=1}^{K}\alpha_{i}p\left(x|\mu_{i},\sigma_{i}^{2}\right)$$

where *p*(*x*| *μ*, *σ*^2^) represents the probability density function for a Gaussian distribution with mean value of *μ* and standard deviation of *σ*. In addition, *α* is the mixture coefficient, $\sum_{i=1}^{K}\alpha_{i}=1$. In our study, we set *K* = 2 assuming that there were two probability distributions modeling the duration of a bathroom visit for a urination and a defecation respectively. We used the Expectation-Maximization (EM) algorithm (Eq. (3)) for fitting the model by maximizing the likelihood function:

(3) $$p\left(x\right)=\prod_{i=1}^{N}p\left(x_{i}\right)$$

where *N* is the number of data points. Python and scikit-learn package were used for the implementation (Pedregosa et al., 2011).

### 2.7 TN separation efficiency model

Wastewater generation patterns from the urine and feces scales were used to model the TN separation efficiency, η_predicted_. We adopted the following procedure:

1. Measure Volume increase and determine event type. For each day when an aggregated TN value was measured by a chemical assay, we examined the signals plots and obtained *u*_i_ and *f*_i_ values during the urination for each *i*-th use event. We used the criteria listed in Supplementary Table S4 and S5 to define urination and hygiene activities and volume increase attributed to water (i.e., washing, flushing) were excluded. The occurrence of a defecation was determined based on temporal duration. For events where urination was not a distinct sub-event, volumes of *u*_i_ = 133 mL urine and *f*_i_ = 80 mL were used; these were obtained from averages of all events from this analysis.
2. Calculate TN (g) mass generated per day. Defining A (g/L) as the concentration of TN in urine, and D (g) as the TN per defecation, we calculated the nitrogen content of both tanks as *TN*_*U*_ = $\sum_{1}^{n}$*u*_*i*_ ∗ *A* and *TN*_*F*_ = $\sum_{1}^{n}$(*f*_*i*_ ∗ *A* + *D*) for each day.
3. Minimize least square. Using the solver function in Microsoft Excel for the A and D parameters, we minimized the difference between calculated and chemically measured ($\sum_{d=1}^{m}\left({\mathrm{TN}}_{U,\mathrm{calc}}-{\left({\mathrm{TN}}_{U,\mathrm{meas}}\right)}^{2}\;\right)\cup$ ($\sum_{d=1}^{m}({\mathrm{TN}}_{F,\mathrm{calc}}-{\left({\mathrm{TN}}_{F,\mathrm{meas}}\right)}^{2}\;$).
4. Calculate predicted daily TN separation. Using the values of A and D from the minimal least square, we calculated $\eta_{\mathrm{pred}}={\mathrm{TN}}_{U}\left(\bar{A},\bar{D}\right)/\left[{\mathrm{TN}}_{U}\left(\bar{A},\bar{D}\right)+{\mathrm{TN}}_{F}\left(\bar{A},\bar{D}\right)\right]$_._

## 3 Results

### 3.1 Baseline characterization of system response and volume separation

Upon installation of the Urine Trap squat plate, the temporal and volume response of the scale measurement system was assessed by pouring known amounts of water (100, 200 and 300 mL) from a bottle with an orifice in the lid to qualitatively emulate urination (Supplementary 2). Separation efficiency, η_vol_ (calculated using Eq. (1)), was in 80–90 % range at a physiological flow rate of 13 mL/s, and decreased to 60 % in the case of large volumes pouring rapidly at 23 mL/s.

Given this volume separation efficiency of the Urine Trap toilet, the expected nutrient separation efficiency was calculated. The normative value of TN distribution in urine and feces per person per day is 11 g in urine and 1.8 g in feces (Rose et al., 2015) (details in Supplementary material 3).

In the ideal case of 100 % of urine going to the urine tank and the feces to the feces tank, the separation efficiency is Ideal η_ΤΝ_ = 11/(11 + 1.8) = 86 %. The measured separation efficiency could be even higher than the ideal 86 % if defecation were rare, as could occur in a workplace toilet. Accounting conservatively for a volume separation efficiency of the Urine Trap of η_vol_ ~ 80 %, the expected separation from this field test was Expected η_ΤΝ_ = Ideal η_ΤΝ_ * η_vol_ ~ 70 %.

### 3.2 Waste stream nutrient content and separation

Nutrient contents and other physico-chemical parameters were measured daily on the two wastewater streams generated by users of the Urine Trap for the three periods of this study.

**Fig. 2:**
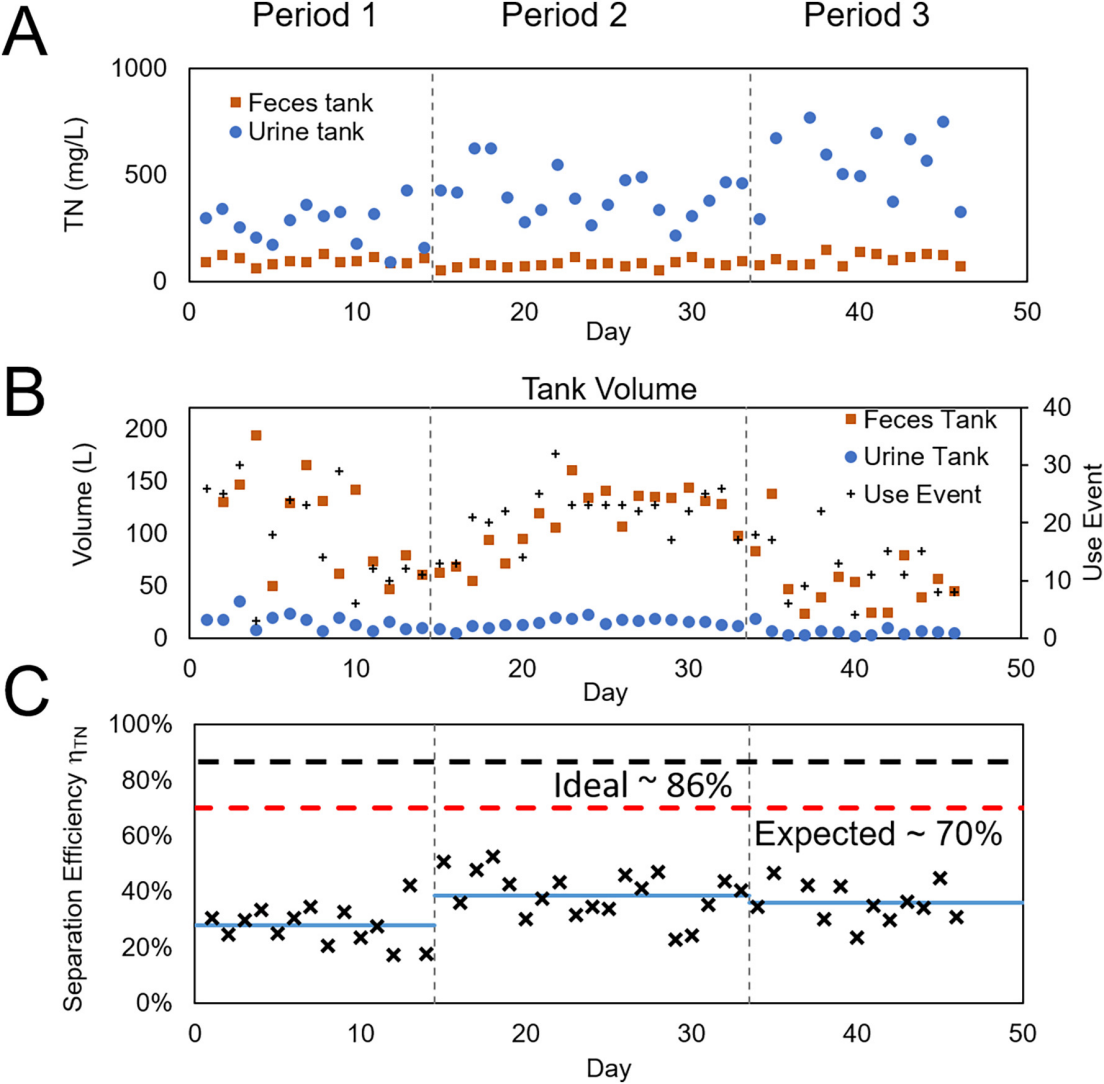
Data was collected in 3 periods indicated by vertical dashed lines: at site A for 14 days (period 1) and after a pause again for 20 days (period 2); site B 16 days (period 3). (A) Total Nitrogen daily values. (B) Daily volume in urine and feces tanks and number of uses. (C) Urine separation efficiency η_ΤΝ_. Blue lines are average values by period from Table 1. Black horizontal line indicates ideal value η_ΤΝ_ ~ 86 % and red line the expected η_ΤΝ_ ~70 %.

The TN values in the urine tank (average TN_U_ = 435 mg/L) were found to be consistently higher than in the feces tank TN_F_ = 92 mg/L (Fig. 2A), with a wide range of daily variability. Since urine TN content is in the range 4000–14,000 mg/L (Rose et al., 2015), TN_U_ = 435 mg/L suggests that urine in the urine tank was diluted by wash water entering this waste stream by a factor of 10 to 30.

The volume of the feces tanks ranged from 60 to 160 L per day, while the urine tank collected a much smaller volume of 6 to 23 L/day (Fig. 2B), corresponding to a volume separation efficiency of η_vol_ = (89 ± 2)%, (*n* = 50) consistent across sites and periods. This high volume separation illustrates that the liquid separation of the Urine Trap squat plate was effective and indicates that only a small fraction of flush water entered the urine tank. The volume separation efficiency η_vol_ does not correlate with the TN separation efficiency η_TN_, since the latter depends on the amount of urine that enters in the feces tank.

Fig. 2C illustrates the separation efficiency η_TN_ calculated from the values of Fig. 2A and B and results in an overall separation efficiency of 36 %.

Table 1 contains the average values for nutrient separation efficiency measured in this study by site and period over a total of 50 days of data collection.

**Table 1:** Nutrient separation efficiency η (Ave ± S.D.) measured by site and study period, from daily values for a total of *n* = 50 days.

Time	Site	Days/uses	η_ΤΝ_ (%)	η_ΤΝ_ (%) supernatant	η_ΤP_ (%)	η_ΤP_ (%) supernatant
Period 1	A	14/243	28 ± 7	38 ± 7	NA	NA
Period 2	A	20/422	41 ± 8	50 ± 7	17 ± 3	21 ± 5
Period 3	B	16/176	40 ± 10	48 ± 10	18 ± 11	23 ± 11
Overall	–	50/841	36 ± 7	45 ± 6	18 ± 1	22 ± 1

The measured η_TN_ = 36 % shows that a completely passive Urine Trap toilet can remove a third of the nitrogen content from the waste stream and provide a jumpstart to onsite wastewater treatment. As a reference, the recently introduced ISO30500 standard for non-sewered sanitation system indicates 70 % TN removal as the target for an adequate wastewater treatment process (ISO30500, 2018).

This study also measured nutrient content data for the feces tank supernatant because it was relevant to practical applications of the Urine Trap system. Several emerging onsite treatment technologies treat the supernatant after settling (Trotochaud et al., 2020; Castro et al., 2021) and thus the supernatant properties downstream from a Urine Trap toilet are relevant as input for such technologies. Despite the fact that these measurements are less rigorous than the homogenized tanks due to the effect of sampling depth, the supernatant separation efficiency (on average η_TN_sup_ = 45 %) was consistently higher than the one obtained from the homogenized feces tanks. We attribute this outcome to the nitrogen content of feces and the fact that the supernatant is expected to have lower fecal content since the tank was left to settle overnight and settled solids contain nitrogen.

Table 1 reports values for a second nutrient of environmental importance, Total Phosphorus (TP). While nitrogen is excreted mostly in urine, TP has similar excretion in urine and feces so a lower reduction in effluent load was expected. Using the midpoint of the ranges from Table S3, ideal η_TP_ = 36 % and expected η_TP_ ~ 36 % ∗ 0.8 = 29 % were calculated and η_TP_ = 18 % was measured.

While a degree of daily variability is expected in the measured nutrient content, we note that there were systematic differences between period 1 and period 2 TN data, which were collected by the same system on the same site. Such a difference was interpreted as the difference in the pool of users and their hygiene practices and was the motivation to seek data from a different pool of users at site B, which yielded similar results to period 2. We hypothesized that hygiene practices are also the cause for the η_ΤΝ_ and η_TP_ to be approximately half of the values we expected, suggesting that, in addition to wash water entering the urine tanks and diluting the TN, a significant amount of urine also ends up in the feces tank.

In order to interpret the performance of the Urine Trap under user test, we examined the temporal data from the digital scales to gain insights into hygiene practices.

### 3.3 Temporal dynamics of waste streams during toilet use

Because of the large amount of data from recordings from digital scales at 1 Hz resolution for the number of toilet uses recorded in this study, a python script described in Methods was developed to automatically extract the section of the data time series corresponding to each use of the toilet. Developing such a method was associated with challenges because of the multiplicity of subevents that may take place during a toilet use including use of spray wand, mug and bucket for hygiene purposes in addition to physiological excretion Supplementary S2).

To facilitate the interpretation of wastewater generation patterns and importantly to determine the start and end time of an individual use of the bathroom, temporal data from the water flow sensor was evaluated. Water sensor data was found to be not useful, because the water line was used both for self-washing and for filling the bucket for flushing, resulting in non-specific signals. In period 3 of this study, the bathroom door opening and closing data from the door switch became available and it was used as a reference and ground truth. In period 3, 176 toilet use events were recorded according to the door sensor signal.

For all periods, the automatic event identification method relied only on the signal of the scales and on empirically defined thresholds.

The accuracy of the start/end times of bathroom use identified by the script was verified against raw data; the event count for period 3 by the script was of 165 events against a reference value of 176 events, an error of 6 %. The automatic count excluded events of short duration below a low cutoff of 15 s, which were interpreted as a mug pour and not as a physiological event.

**Fig. 3:**
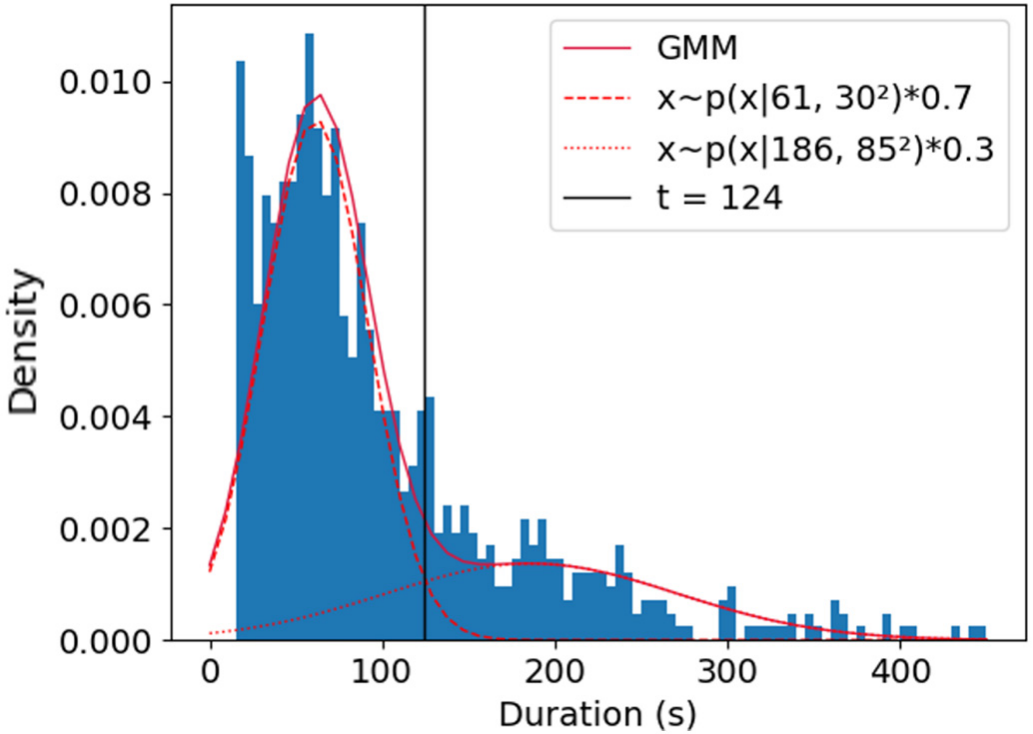
Histogram of duration of toilet use events for all the data (3 periods) of this study. The two Gaussian distributions from the Gaussian Mixed Model (GMM) fit represent: urination for mean duration of 61 s and defecation for mean duration 186 s. The intersection of the curves at *t* = 124 s is the threshold used to classify an event as defecation.

The automatic event identification method was used to obtain the number of bathroom visits for period 1 and 2 of the study in Table 1. This has a total of 841 events using the value of 176 uses from period 3 determined by the door sensor, or 830 events using the automatic event identification method for all periods and 165 uses for period 3. The duration for each event was plotted as a histogram in Fig. 3. The histogram excludes a handful of outlier events longer than 450 s.

Examining the histogram in Fig. 3, we expected to find a bimodal distribution of duration based on the two physiological events of urination and defecation, with the latter being longer. Using the GMM model as described in the Methods Section 2.6, we found two Gaussian distributions with (mean, standard deviation) as (61, 30) and (186, 85) representing urine and defecation events, respectively. The Probability density functions *p*_*u*_ for the urination event and *p*_*e*_ for the defecation event are shown in Fig. 3. We found a threshold *t* = 124 s, where *p*_*u*_^*t*^ < *p*_*e*_^*t*^ *if t* ≤ 124 and *p*_*u*_^*t*^ > *p*_*e*_^*t*^ *if t* > 124. Note that 25 % of the events out of the 830 total number of events in this study were above the threshold and therefore classified as a defecation.

### 3.4 Wastewater streams patterns

Having segmented the data into toilet use events, the pattern of waste streams within an event was examined to gain insights into subevents and hygiene practices.

**Fig. 4:**
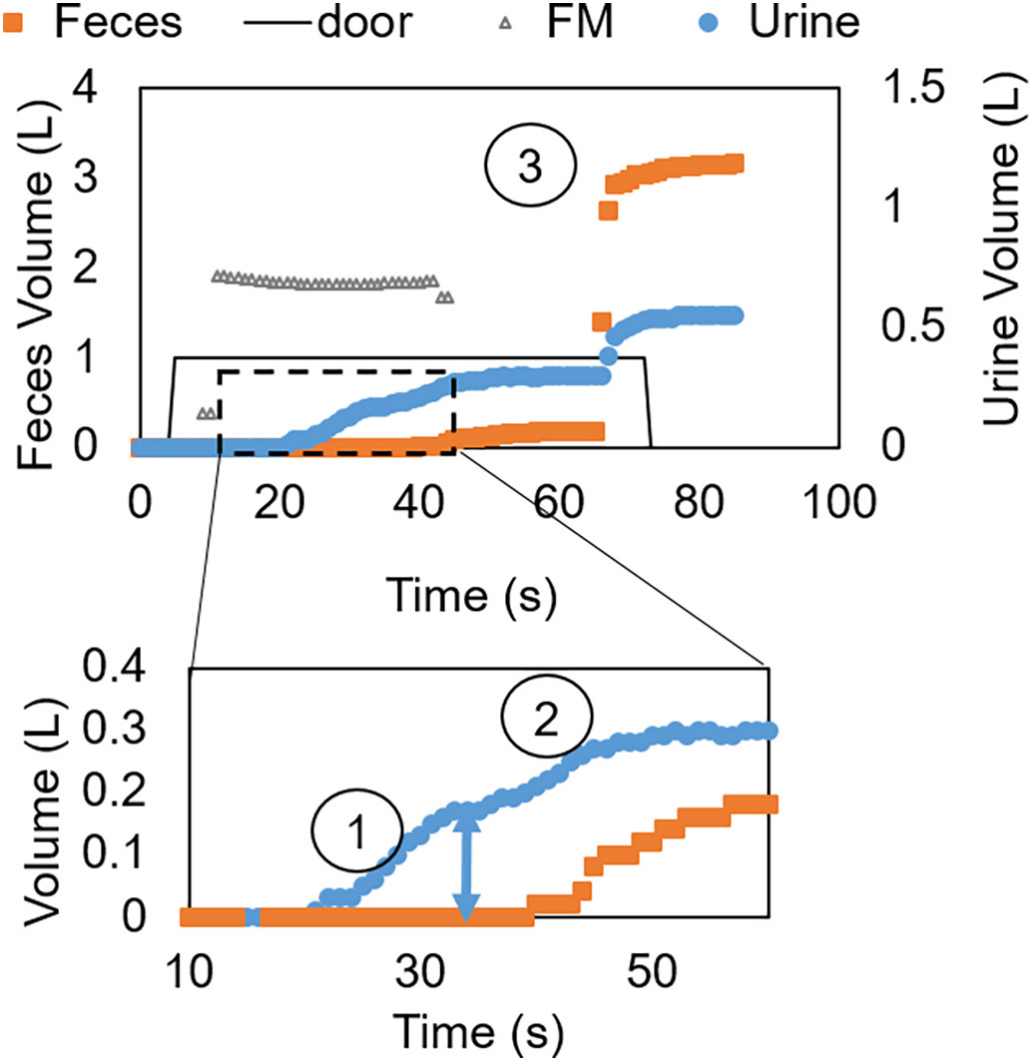
Sensor data illustrating a urination event. After the user enters the bathroom, tap water starts to fill out a bucket, followed by 1.Urination 2. Cleansing 3. Bucket flush. The arrow segment in the insert indicates the volume ΔU attributed to urine.

Fig. 4 illustrates urination-event time series data collected by the four sensors: volume of UT and FT, water use, and door open/close. The data in Fig. 4 was interpreted as the following: the user entered the bathroom and closed the door, the water tap was opened to fill the bucket, then urination occurred and the squat plate directed the liquid to the urine tank, then at time t ~ 40 s, wash water was used, followed by a bucket flush. The volume of urine is estimated to be approximately 200 mL (double arrow in Fig. 4 insert), and the overall volume of liquid in the urine tank is 500 mL, illustrating the wash water dilution of urine as reflected by TN values in the urine tank.

To distinguish the user action of cleansing and flushing from physiological excretion, a series of tests were conducted to measure system response to simulated urination and to hygiene activities (spraying, mug pouring, bucket pouring). The measured signal patterns over replicated tests were used to identify characteristic flow rates (described in Supplementary 2) and were the source for criteria used for classification summarized in Supplementary Table S5).

**Fig. 5:**
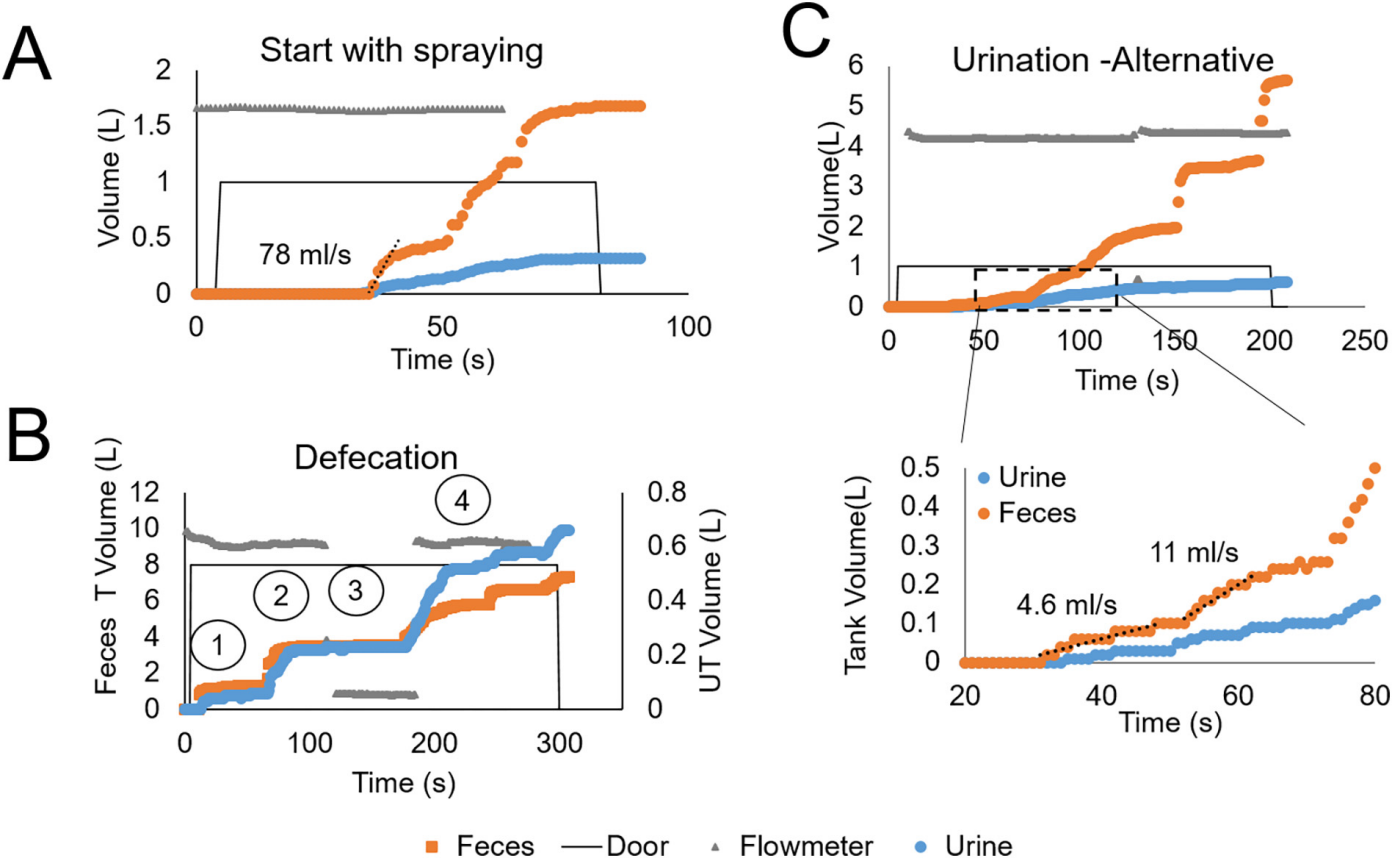
Wastewater volume over time during a toilet use as defined by door close position. Flowmeter values are shown as a.u. (A) Spray/Pour first, unclear when a physiological event occurs. (B) 1. a mug is poured, 2. urination, 3. pause attributed to defecation, 4. multiple flushes. (C) Alternative urination: liquid at physiological flow rate went mostly in the feces tank.

The event in Fig. 4 suggests that all the TN from urine is in the urine tank, because the feces tank signal did not increase: this was the scenario the Urine Trap squat plate was designed for. However, we found that many wastewater patterns were different from this case as illustrated in Fig. 5.

Fig. 5A indicates that wastewater generation started with water spraying, recognized by its high, non-physiological flow rate: this hygiene practice improves cleaning of the toilet after use; unfortunately, it degrades the teapot effect that the Urine Trap separation is based on and causes urine and its nutrients to distribute across waste streams. Fig. 5B was interpreted as a defecation because of its long duration and various mug pours as labelled by the numbers in the figure. Fig. 5C illustrates a urination that ends up mostly in the feces tank. We believe this pattern may be caused by different body positions of the user or by deliberately choosing to aim at the feces hole instead of the flat ceramic portion of the plate; we labelled this pattern as urination – alternative. The flow meter data was also reported in all panels of Fig. 5 to illustrate its poor discriminatory value since the water was essentially running for most of the toilet visit.

Using the criteria of Table S5, all the data in period 3 and a subset of the data in period 1 and 2 were analyzed for classification purposes, for a total of 462 toilet uses. The number and percentage of subevent types that impact nitrogen separation are listed in Table 2.

**Table 2:** Descriptive statistics of sub-events classified according to the criteria developed in this study for 462 bathroom visits.

Event type	Period 1 (28 % η_ΤΝ_)		Period 2 (41 % η_ΤΝ_)		Period 3 (40 % η_ΤΝ_)	
	Number	Percentage	Number	Percentage	Number	Percentage
Bathroom visit	119	100 %	167	100 %	176	100 %
Urination - first	26	22 %	45	27 %	81	46 %
Alternative urination	13	11 %	35	21 %	51	29 %
Start with Spray/pour	80	67 %	87	52 %	44	25 %
Defecation^a^	34	29 %	61	37 %	52	30 %

a Not mutually exclusive to any of the other three event types.

A striking result from Table 2 is that only a fraction (22 % to 46 %) of toilet uses starts with urination. The practice of cleaning the toilet bowl while using the toilet is frequent and particularly common in period 1, the data set for which the η_ΤΝ_ is lowest. Such practice alone does not impact nitrogen separation; however, nitrogen separation is affected if it takes place while the user urinates. There is also a considerable number of events that we classified as urination in alternative positions: this is an important parameter to track because it could be impacted by an education campaign for users in future studies. Finally, the percentage of defecation events (29 % to 37 %) was found to be higher than normative values for the number of urination/defecation of a person per day (the number is 6/1, assuming that one urination also occurs when a defecation occurs (Rose et al., 2015) see Supplementary 3). It is possible that our classification overestimates the number of defecations since our criteria relies on duration, and multiple washes can also occur if the user does not feel well or is menstruating. Nonetheless, there were defecations as indicated by the very high TSS values in the feces tank and by the color of the sample (Supplementary Fig. S1.1B). Because studies relying on the workplace toilet often capture mostly urine, we believe the observation of defecations strengthens the case for this study to be representative of system performance in all settings, not only public toilet settings.

### 3.5 Modeling TN separation efficiency

The subevent analysis in our study is most reliable for the 16 days of period 3, where the door signal provided a valuable reference. For this subset of 176 events, we attempted to use volume data attributed to urination and D values to model the η_ΤΝ_ measured for the 16 days of this period.

**Fig. 6:**
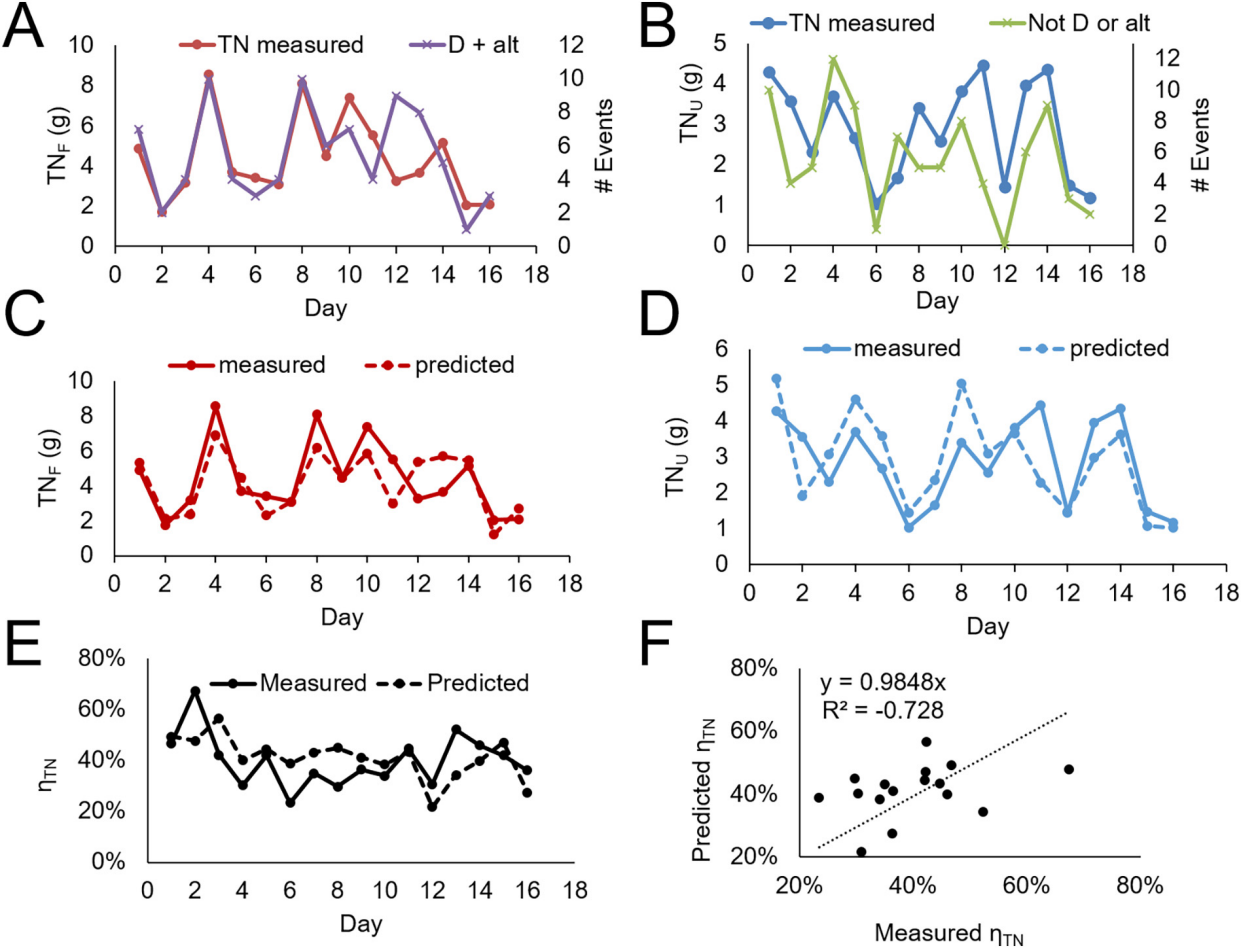
Modeled vs predicted daily TN values for 16 days in period 3. Measured daily values TN_F_ (A) and TN_U_ (B) relative to number of subevents. (C) and (D) TN amount measured and predicted by waste stream. (E) η_TN_ predicted and measured by day. (F) predicted η_TN_ plotted against measured η_TN_ and linear fit.

From the TN concentration and tank volume, the amount in grams of TN_U_ and TN_F_ was calculated and is shown in Fig. 6 A-B. We observe that the daily variation TN_F_ correlated well with the number of subevents equal to the sum of D and alternative urinations, while TN_U_ correlates well with the number of urination – first.

The predicted TN_F_ and TN_U_ were obtained as described by manually assessing volumes ascribed to urination in each event and by fitting the parameters of A = C_TN_ in urine = 1.98 g/L and D = 0.7 g TN for a defecation event. Using these values, the predicted TN_F_ and TN_U_ amounts approximate well the measured values (Fig. 6 C–D).

The value of the parameter A = 1.98 g/L is below the range reported in the literature (4–14 g/L) (Rose et al., 2015), and so is the case for D = 0.7 g/event, given the range 0.8–4.5 g TN/defecation reported in the literature (see Table S3) (Rose et al., 2015). This suggests that our wastewater pattern analysis overestimated both the volume and number of the respective physiological events, and water was poured when we assumed a physiological excretion. Nonetheless the wastewater volume patterns obtained by the sensors enable the predicted η_ΤΝ_ to describe accurately the measured η_ΤΝ_ (Fig. 6E) and a linear fit between the two is shown in Fig. 6F. Overall, this model supports our interpretation that hygiene practices and behavior impact the separation efficiency.

## 4 Discussion

This study conducted user testing of the Urine Trap toilet in a relevant environment with women workers who had unmonitored access to the system. The study recorded as many as 841 uses collecting over two sites in an urban region of southern India and in three different periods, thus we believe that our results are representative for this setting.

The Urine Trap appeared like a regular squat plate, thus it did not change the user experience and operated completely passively, not requiring any electricity. No maintenance of any kind was required on the squat plate during the period of the study. The study determined the separation efficiency by nutrients (TN, TP) and found that the Urine Trap toilet technology enables substantial separation of nitrogen from the wastewater and some removal of phosphorus. The average removal was 36 % of nitrogen from the waste stream, or 45 % for supernatant and these values represent a valuable reduction that may ease the operation of onsite wastewater treatment benchmarked by ISO30500 to removing 70 % of the influent TN.

While effective, the separation efficiency of the Urine Trap squat plate was significantly lower than the 70 % TN removal expected based on engineering characterization. This observation underlines the importance of field testing in relevant settings and drove the development of a method to gain insights into user behavior that may impact system performance.

By instrumenting the wastewater collection tanks and bathroom stall door and water line with sensors and recording with high temporal resolution, we demonstrated a method to non-invasively yet quantitatively gain insights into user behavior in a private act such as toilet use.

Facilitated by automation in various aspects of the analysis, the examination of this dynamic dataset provided information on user actions and practices. Informed by physiological parameters of urination and by the characterization of the measurement system, this study revealed the ability to identify type of excretion and user washing habits and, importantly, the timing of it, by using the two tank volumes. The door closure data was found to be a valuable reference point, the water use meter was not. The results showed that habits such as wetting the squat plate before use was common. While this practice per se does not impact nitrogen separation, if this is carried out while urinating, it degrades the nitrogen separation. Also, the data shows that body position or aiming during urination has a much wider range of possibilities than originally envisioned.

A limitation of this study is that subevent classification relied on criteria developed during “mock uses” of the toilet and was not verified against user survey. Another limitation was the use of empirically defined thresholds to define specific patterns of use. This paper describes the rationale for the choices and our team evaluated the robustness of the results, examining values around the selected thresholds; however, we recognize that there is a degree of arbitrariness in these definitions. We suspect that we overestimated some of the reported results, possibly the number of defecations. Nonetheless the examination of the wastewater pattern and event classification allows to reasonably model the nitrogen values measured in the two waste streams.

The large dataset produced by this study has not been fully explored for this manuscript and will be made available to a public depository for others to explore.

This evaluation of the Urine Trap flush toilet in South Asia yielded several learnings that inform next steps.

While urine-separation toilets are largely unexplored in washing cultures, our results suggest that source-separation can be implemented in these settings despite personal wash water use creating challenges.

As informed by these experimental results, enhancement to the separation performance of the Urine Trap system are being explored by selecting different materials/coatings for the toilet.

This team is also planning to conduct a survey of users with a detailed questionnaire to validate the user actions and preferences identified from sensor data.

The present study was an engineering evaluation that did not include any user awareness of the technology. Prior studies on acceptance of source-separating sanitation systems have suggested that public education is needed to increase awareness and build trust with consumers (Lamichhane and Babcock, 2013; Simha et al., 2021).

As next steps, we plan to evaluate how user education may improve separation performance with minimal impact on the user experience. For example, we will test toilet-aiming stickers that are commercially available for public urinals with the goal of reducing the occurrence of non-ergonomic body position in urination, such as aiming at the back-exit (feces) hole.

## 5 Conclusion

This study conducted a field-testing evaluation of a novel design for a urine-diversion flush toilet in a women's bathroom with personal wash water. This passive system operated for over 841 uses without any need for maintenance and collected a high nitrogen concentration liquid (>400 mg/L) in the urine tank. The Urine Trap was effective in removing approximately one third of nitrogen from the wastewater (36 % nitrogen separation efficiency). Engineering characterization indicated that the Urine Trap can achieve as high as 70–80 % nitrogen separation that was not observed in this field testing due to user hygiene practices.

By instrumenting the wastewater collection tanks and bathroom fixtures with commercial sensors, this study demonstrated a method to gain insights into user hygiene practices. This sensor-based evaluation approach and associated computational code for analysis provide a valuable tool for non-invasively yet quantitatively analyzing the impact of user behavior in sanitation-related studies.

Adoption of urine diversion solutions for nitrogen separation will be greatly beneficial to the environment and increase sustainability, and learnings from evaluation in relevant settings will inform technology development and deployment strategies to maximize performance of source-separating toilets.

## CRediT authorship contribution statement

**Prateek Kachoria:** Investigation, Software, Formal analysis. **Sarani Sasidaran:** Investigation, Formal analysis. **Claire M. Welling:** Formal analysis, Data curation, Visualization, Writing – review & editing. **Praveen Rosario:** Resources, Formal analysis. **Jin Zhou:** Software, Data curation, Formal analysis. **Krishnendu Chakrabarty:** Supervision, Writing – review & editing. **Harald Gründl:** Conceptualization, Resources. **Lotte Kristoferitsch:** Investigation, Resources. **Sonia Grego:** Methodology, Project administration, Visualization, Writing – original draft.

## Declaration of competing interest

The authors declare the following financial interests/personal relationships which may be considered as potential competing interests:

HG and LK were employees of EOOS a company assigned a patent on the Urine Trap technology and pursuing its commercialization.

## Data Availability

The code generated in this study is available open access at github.com/code-by-jin/EOOS-Data-Analysis. The dataset generated by this study is available under CC BY 4.0 at 10.6084/m9.figshare.20240025.

## Appendix A Supplementary data

Supplementary material Image 1
