# Supplementary material for: Sensor-based evaluation of a Urine Trap toilet in a shared bathroom
Source: Sci Total Environ. 2023 Jan 20;857:159178. doi: 10.1016/j.scitotenv.2022.159178 (PMC9742848; doi:10.1016/j.scitotenv.2022.159178)
Supplement: Supplementary file 1 — Supplementary material [file mmc1.docx]

# Appendix A: Supplementary Material

Table of contents

**Supplementary S1:** Experimental Apparatus pictures

**Supplementary S2**: Characterization of plumbing and system response

**Supplementary S3**: Nitrogen content in urine and feces

**Supplementary S4:** Physiological urination parameters from literature

**Supplementary S5:** Criteria for wastewater volume pattern classification

**Supplementary S6:** Wastewater quality parameters in urine tank and in feces tank (both macerated and supernatant)

## Supplementary S1 Experimental Apparatus

| 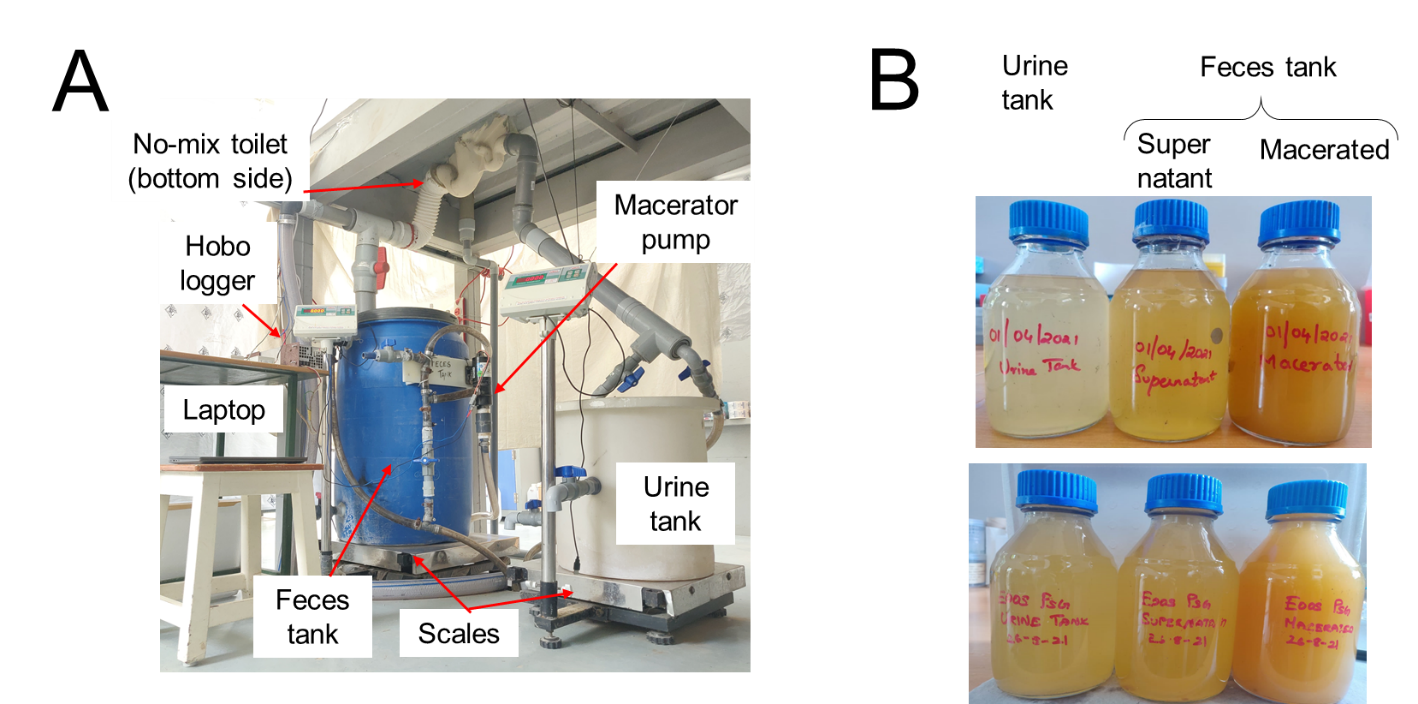 |
| --- |
| Figure S1.1 A. Measurement apparatus. B. Two examples of typical appearance of wastewater samples |

| 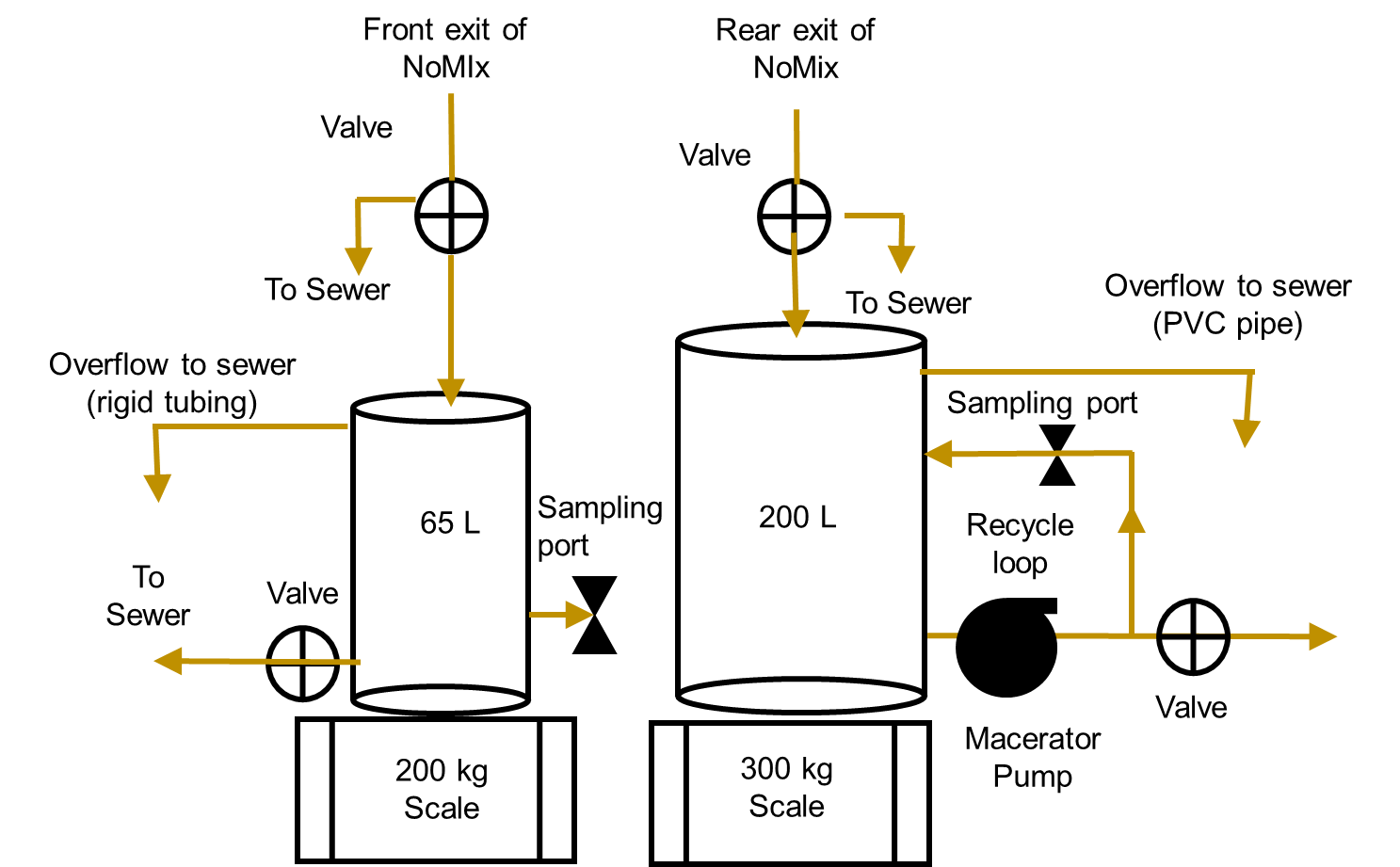 |
| --- |
| Figure S1.2 Detailed schematic of the scale measurement apparatus |

## Supplementary S2 Characterization of measurement system response

A multiplicity of subevents may take place during a toilet use as summarized in table S2.1 and the measurement system response to these was characterized as follows.

**Table S2.1.** Description of the subevents that may take place during one event of toilet use.

| Subevents | Description and timing |
| --- | --- |
| Physiological excretion | 1. Urination 2. Defecation   Typically, urination occurring first. Defecation may or may not occur. |
| Use of spray wand | 1. After physiological excretion for cleansing 2. Before excretion, in order to wet the squat plate surface, to ease its cleaning   Water use is recorded by the flow meter simultaneously to an increase in tank weight |
| Use of mug | 1. After excretion, for cleansing 2. Before excretion, to wet the squat plate surface, to ease its cleaning 3. After excretion and cleansing, for flushing |
| Use of bucket | For flushing. |

**System response to urination**

Known amounts of water (100, 200 and 300 ml) were repeatedly poured on the squat plate from a bottle with an orifice in the lid to qualitatively emulate urination. The water was poured pointing at the ceramic portion of the plate from a squatting position emulating the user position when voiding. Note that F is the slope of the feces tank scale reading and U is the slope of the urine tank scale reading.

The majority of the input volume went to the urine tank (Figure S2.1 A). The measured volume increase ΔF+ΔU was typically 20-40 ml higher or lower than the input volume: we interpreted this as volume stored in the S-trap that reached the tank at the subsequent toilet use.

The Separation efficiency by volume η_vol_ was calculated by dividing by scale reading of ΔF+ΔU and by dividing by the poured volume (Figure S2.1 B). The 300 ml volume was poured also at a “fast” 23 ml/s rate, which is an upper limit physiological value. Separation efficiency η_vol_ was above 80% and approximately 90% range for most cases, decreasing to 60% in the case of large volumes pouring rapidly.

The value of η_vol_ =80% was selected for estimates in the manuscript.

| 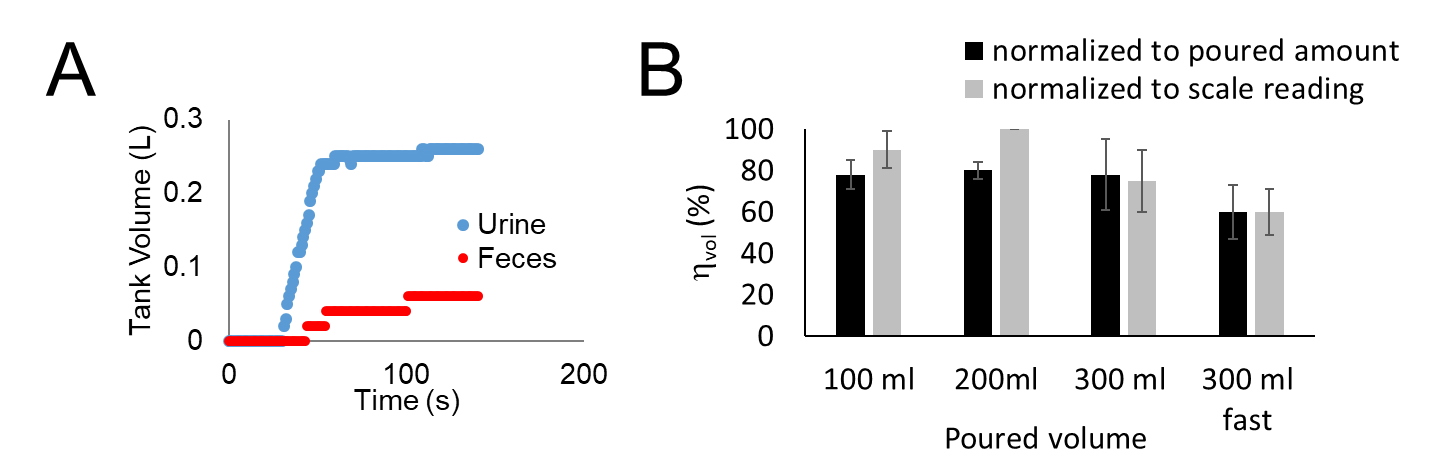 |
| --- |
| Figure S2.1 A. example of system response to urination separation B. Volume separation efficiency for urination simulation 100 ml (n=10 tests), 200 ml (n=4), 300 ml (n=9, n=10 fast) |

**Delays introduced by measurement system.**

The rate at which liquid was recorded by the scales was an important parameter in this study because it was used to distinguish between urination at physiological rates and water pouring. We evaluate the rate dampening introduced by the scale by comparing the time required for pouring a 300 ml solution and the rate of urine tank volume change. We found that, for the urine tank, the dampening introduced by the system is negligible (~ 1.4% rate change). On the other hand, the feces tank scale reading had a 20% temporal variation over pouring time (either increased or decreased) due to the large volume P-trap. Importantly, we noted that the urine tank always moved sooner relative to the feces tank by an average of 7 ± 3 seconds (n=19 tests with 300 ml) while simulating urination. This observation was a key criterion to classify urination at the start of toilet use event.

**System response to pouring of mugs and buckets and to spray washing**

Mug flushing: a mug (1 L maximum capacity) was available in the bathroom stall to perform personal hygiene. We measured the system response to mug pour of 500 ml and 1000 ml in eight separate tests. The average measured rate was 109 ± 46 ml/sec (n=8) with the smallest signal slope of 35 ml/s for a short period of less than 10 seconds. (Figure S2.2A) Thus, the criteria assigned to recognize a mug pour was a volume change between 500 and 1000 ml, poured at a speed of at least 35 ml/s (500 ml in 5 seconds was typical).

Bucket flush: a 5 liter capacity bucket was available in the stall for toilet flushing. Data was collected on 3 separate experiments in two sites, pouring with a bucket volume of 1.5L, 2L and 4 L. The average duration of the event recorded by the feces tank was t=9± 3 sec (n=10) for the scale signal to reach 90% of the asymptote (Figure S2.2B). The slope depended on poured volume and ranged from 128 to 728 ml/sec (n=10). Thus we defined the criteria identifying a bucket flush as an abrupt volume change of at least 2L in 16 seconds (equivalent F > 125 ml/s).

Spray faucet: The experimentally measured system response to use of the spray faucet emulating cleaning of the bowl was different by site, depending on the water pressure of the faucet. At Site A, it was F = 32 ± 12 ml/sec (n=10), and at site B, it was higher at F = 75± 15 ml/sec (n=12) (Figure S2.2C).

| 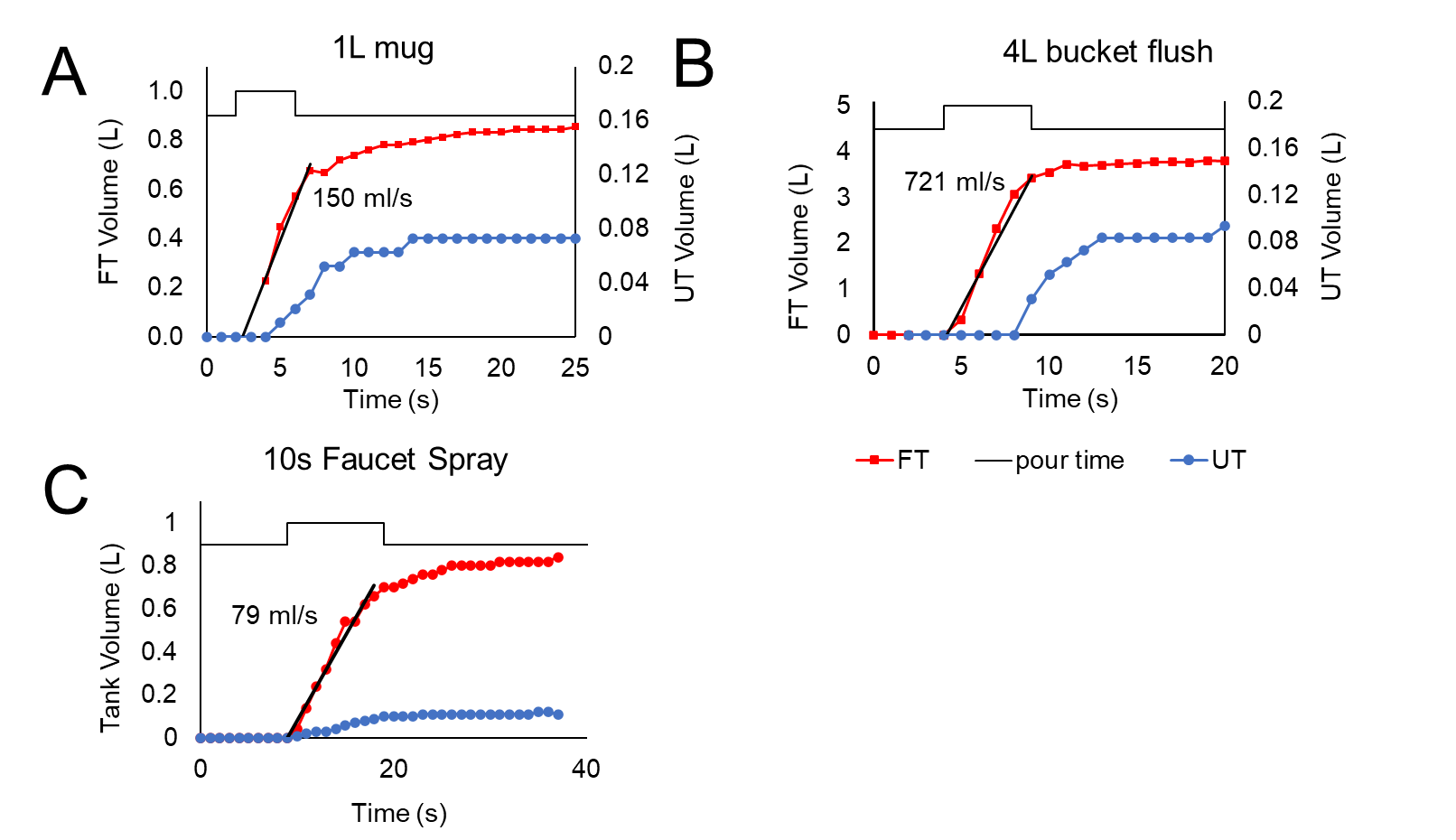 |
| --- |
| Figure S2.2. System response to A. bucket flush, B. mug flush, and C. spray faucet at site B. |

## Supplementary S3: Nitrogen content in urine and feces

Rose and co-authors conducted a thorough review of the generation rate and composition of the solid and liquid fraction of human excreta from 95 studies from all over the globe (Rose et al. 2015) and reported median values, 25-75% percentile range and min-max of the range of relevant parameter (Table S3).

| **Table S3** Summary of human feces and urine characteristics from (Rose et al. 2015). | | | |
| --- | --- | --- | --- |
| Parameter | Urine | Feces | Data source in Rose et al |
| TN daily generation | 11 g/cap/day  (2-35 g/cap/day) | 1.8 g/cap/day  (0.9-4.9 g/cap/day) | Table 12 and page 1842 |
| TN concentration | 4-14 g/L | - | Table 9 |
| TN amount excreted per event | 2 g  (0.9 -3.3) g range | 1.6 g  (0.8-4.5) g range | Calculated from data in this table |
| TP generation | 0.45- 1.3 g/cap/day | 0.35-2.7 g/cap/day | Table 8 (urine) Table 5 (feces) |
| Amount generation | 1.4 L/cap/day | 128 g/cap/day | Table 12, median values |
| Frequency | 6/day | 1.1/day | Table 12 |
|  | | | |

## Supplementary S4: physiological urination parameters from the literature

Uroflowmetry is the measurement of urine flow over time and it is a baseline medical test for evaluating urinary dysfunction in men and in women. Normative studies developed to establish a healthy baseline for both men and women for medical purposes have been conducted. We reviewed those studies to select a range of womens’ physiological urine flow rates, voiding volume and voiding time to guide our data analysis.

We report in table S4 data from a global review study (Sorel et al. 2017) as well as multiple studies conducted on Indian women in a squatting position, the same used in this study (Choudhary et al. 2011) (Singla et al. 2018) (Gupta et al. 2008). The peak flow rate during one voiding event is of medical relevance and depends on voiding volume. The average flow rate values were used in our study.

| **Table S4:** literature data related to urine volume, flow rate and duration. Average ± S.D. | | | | | |
| --- | --- | --- | --- | --- | --- |
| volume (ml) | Peak flow rate (ml/s) | Average flow rate  ml/s | Voiding time (s) | reference | Notes |
| 338 ± 161 | 23.5 ± 10 | 13.1 ± 6 | 29 ± 17 | Sorel 2017 | Global review, women values |
| 294 ± 159 ml | 23.7 ± 9.0 | 13.1 ± 5.3 |  | Choudhari 2011 | Indian asymptomatic women in squatting positon |
| 259 ± 70 ml | 24.8 ± 4.9 | 12.3 ± 3.3 |  | Gupta 2006 | Indian asymptomatic women in squatting positon |
| 400 ± 190 | 22 ± 8 | 12 ± 5 | 36 ± 17 | Singla 2018 | Indian premenopausal (average age 33) women |

The range of physiological values was defined (upper bound) = max (average + S.D) and (lower bound)= min (average – S.D) from the values in table S4.

In summary, physiological urination parameters for this study (rounded for convenience) are:

- Urine Volume = 15 – 500 ml
- Voiding time = 12 – 50 sec
- Flow rate = average 13 ml/sec, range 7 – 19 ml/sec

**References**

Choudhary, M., M. Agarwal, A. Mandal, R. Mavuduru and S. Singh (2011). "A Study Of Normative Flow-Volume Relations In Healthy Indian Women In Squatting Voiding Position: Reappraisal Of Applicability Of Liverpool Nomograms In This Population." European Urology Supplements **2**(10): 288.

Gupta, N. P., A. Kumar and R. Kumar (2008). "Does position affect uroflowmetry parameters in women?" Urologia internationalis **80**(1): 37-40.

Rose, C., A. Parker, B. Jefferson and E. Cartmell (2015). "The characterization of feces and urine: a review of the literature to inform advanced treatment technology." Critical reviews in environmental science and technology **45**(17): 1827-1879.

Singla, D., P. Malik, M. Sangwan, M. Garg and D. Bansal (2018). "Age, gender, and voided volume dependency of peak urinary flow rate and uroflowmetry nomogram in a tertiary care centre." Asian Pacific Journal of Health Sciences **5**(2): 13-16.

Sorel, M. R., H. J. Reitsma, P. F. Rosier, R. J. Bosch and L. M. de Kort (2017). "Uroflowmetry in healthy women: A systematic review." Neurourology and urodynamics **36**(4): 953-959.

| Table S5: excretion or hygiene event types classified based on wastewater volume generation patterns. Each criteria condition in one category must be met. ΔU: urine tank volume change; ΔF: feces tank volume change, F: slope in the feces tank; U: slope in the feces tank urine, T: time interval. | | |
| --- | --- | --- |
| Type | Criteria | Justification |
| Urination  At the start | U moves before F  ΔU > ΔF  10 ml < (ΔU + ΔF) < 500 ml  5 s < T <50 s | System response characteristic – suppl. S2B  Squat plate urine separation  500 ml max Physiological range for urine  50 s is max physiological duration.  12 s is min physiological duration however pouring may occur after 5 seconds and overlap with it |
| Alternative urination | F< 20 ml/s  (ΔU + ΔF) <500ml, T <40s  ΔU < ΔF (eta <50%) | Liquid at physiological rate (<20 ml/sec) reaches the feces tank, indicating urination (max volume 500 ml, and 40 s duration) aiming at or reaching the feces hole of the squat plate. |
| Defecation | T > 124 sec  ΔF > 2 L  ≥3 sub-events | Duration cut-off obtained from histogram analysis.  High volume due to requirement for flushing feces.  Multiple changes in slopes and sub-event because self-cleaning is more complex. |
| Spray faucet | 10 ml/s < F < 30 ml/s at site A, F > 50 ml/sec at site B  ΔU + ΔF > 500ml  Flowmeter ON | The rate is slightly higher than urination at site A, and much higher than physiological at site B  Volume is over the physiological volume max. |
| Mug flush | F > 60 ml/s slope  500 mL < ΔU + ΔF <1000 ml | Slope in feces tank when pouring much higher than spraying at site A. Volume is over physiological urination limit. |
| Bucket Flush | ΔF > 2 L (and up to 4 L)  T ≤ 16 s | Measured bucket pouring response. |

## Supplementary S5 Criteria for wastewater volume pattern classification

## Supplementary S6: Wastewater quality parameters in urine tank and in feces tank (both macerated and supernatant)

**Table S6.1 Wastewater** quality parameters average (St. dev.) for urine and macerated feces tank.

|  | Urine | | | | Feces (macerated) | | | | η (%) |
| --- | --- | --- | --- | --- | --- | --- | --- | --- | --- |
| mg/L | Period 1 | Period 2 | Period 3 | All | Period 1 | Period 2 | Period 3 | All | **All** |
| TN | 265 (93) | 425 (114) | 623 (213) | 435 (203) | 97 (19) | 80 (16) | 103 (28) | 92 (23) | **36** |
| NH3 | 234 (93) | 381 (128) | 537 (219) | 383 (192) | 45 (14) | 45 (9) | 44 (10) | 45 (11) | **49** |
| TP | - | 69 (16) | 77 (36) | 71 (27) | - | 40 (8) | 50 (16) | 44 (13) | **17** |
| COD | 552 (207) | 888 (211) | 1227 (382) | 952 (381) | 2002 (603) | 1003 (275) | 1231 (488) | 1251 (543) | **10** |
| TSS | 184 (103) | 229 (67) | 219 (110) | 217 (86) | 1140 (466) | 426 (122) | 558 (330) | 565 (348) | - |
| TS (g/L) | 3.6 (1.9) | 2.7 (0.5) | 4.8 (1.5) | 3.5 (1.5) | 3.5 (9.9) | 1.9 (0.2) | 3.0 (0.5) | 2.5 (0.8) | - |
| EC (mS/cm) | 4.5 (0.9) | 6.4 (1.2) | 8.1(1.8) | 6.4 (1.9) | 2.7 (0.6) | 2.7 (0.1) | 3.1 (0.1) | 2.9 (0.4) | - |
| Volume (L) | 15 (7.9) | 14 (4.7) | 4.9 (2.3) | 12 (6.8) | 105 (48) | 113 (29) | 44 (18) | 89 (45) | - |

**Table S6.2**  Conventional wastewater with no separated waste stream (n = 3) from site A, period 1 as reference, to compare with Table S6.1 and with the reported calculated values from separated streams. Average (st.dev.) are reported.

|  | Measured (period 1) | Calculated (period 1) |
| --- | --- | --- |
| TN (mg/L) | 130 (32) | 117 (21) |
| NH3 (mg/L) | 81 (29) | 68 (20) |
| COD (mg/L) | 1782 (300) | 1814 (538) |
| TS (g/L) | 2.7 (0.2) | 3.5 (1.1) |
| EC (mS/cm) | 3.2 (0.4) | 3.0 (0.5) |
| Volume (L) | 118 (3) | 120 (56) |

**Table S6.3**  Wastewater quality parameters average (St. Dev.) for urine tank and feces tank supernatant

|  | Urine | | | | Feces (supernatant) | | | | η (%) |
| --- | --- | --- | --- | --- | --- | --- | --- | --- | --- |
| mg/L | Period 1 | Period 2 | Period 3 | All | Period 1 | Period 2 | Period 3 | All | All |
| TN | 265 (93) | 425 (114) | 623 (213) | 435 (203) | 60 (14) | 61 (14) | 74 (18) | 65 (16) | 44 |
| NH3 | 234 (93) | 381 (128) | 537 (219) | 383 (192) | 41 (13) | 44 (9) | 39 (10) | 42 (10) | 51 |
| TP | - | 69 (16) | 77 (36) | 71 (27) | - | 32 (9) | 34 (17) | 33 (13) | 21.5 |
| COD | 552 (207) | 888 (211) | 1227 (382) | 952 (381) | 550 (205) | 491 (149) | 669 (509) | 567 (340) | 18 |
| TSS | 184 (103) | 229 (67) | 219 (110) | 217 (86) | 113 (55) | 98 (54) | 230 (254) | 148 (168) | - |
| TS (g/L) | 3.6 (1.9) | 2.7 (0.5) | 4.8 (1.5) | 3.5 (1.5) | 1.7 (0.7) | 1.5 (0.2) | 2.5 (0.7) | 1.9 (0.6) | - |
| EC (mS/cm) | 4.5 (0.9) | 6.4 (1.2) | 8.1(1.8) | 6.4 (1.9) | 2.6 (0.1) | 2.7 (0.1) | 3.1 (0.1) | - | - |
| Volume (L) | 15.4 (7.9) | 14.2 (4.7) | 4.9 (2.3) | 11.7 (6.8) | 105 (48) | 113 (29) | 44 (18) | 89 (45) |  |
